# Guidance for using artificial intelligence for title and abstract screening while conducting knowledge syntheses

**DOI:** 10.1186/s12874-021-01451-2

**Published:** 2021-12-20

**Authors:** Candyce Hamel, Mona Hersi, Shannon E. Kelly, Andrea C. Tricco, Sharon Straus, George Wells, Ba’ Pham, Brian Hutton

**Affiliations:** 1 Clinical Epidemiology Program, Ottawa Hospital Research Institute, Ottawa, Ontario Canada; 2 Cardiovascular Research Methods Centre, University of Ottawa Heart Institute, Ottawa, Ontario Canada; 3 School of Epidemiology and Public Health, University of Ottawa, Ottawa, Ontario Canada; 4 Knowledge Translation Program, Li Ka Shing Knowledge Institute, St. Michael’s Hospital, Toronto, ON Canada; 5 Epidemiology Division and Institute for Health, Management, and Evaluation, Dalla Lana School of Public Health, University of Toronto, Toronto, Ontario Canada; 6 Department of Medicine, University of Toronto, Toronto, ON Canada

**Keywords:** Artificial intelligence, Active machine-learning, Best practice guidance, Knowledge Synthesis, Prioritization, Title and abstract screening

## Abstract

**Background:**

Systematic reviews are the cornerstone of evidence-based medicine. However, systematic reviews are time consuming and there is growing demand to produce evidence more quickly, while maintaining robust methods. In recent years, artificial intelligence and active-machine learning (AML) have been implemented into several SR software applications. As some of the barriers to adoption of new technologies are the challenges in set-up and how best to use these technologies, we have provided different situations and considerations for knowledge synthesis teams to consider when using artificial intelligence and AML for title and abstract screening.

**Methods:**

We retrospectively evaluated the implementation and performance of AML across a set of ten historically completed systematic reviews. Based upon the findings from this work and in consideration of the barriers we have encountered and navigated during the past 24 months in using these tools prospectively in our research, we discussed and developed a series of practical recommendations for research teams to consider in seeking to implement AML tools for citation screening into their workflow.

**Results:**

We developed a seven-step framework and provide guidance for when and how to integrate artificial intelligence and AML into the title and abstract screening process. Steps include: (1) Consulting with Knowledge user/Expert Panel; (2) Developing the search strategy; (3) Preparing your review team; (4) Preparing your database; (5) Building the initial training set; (6) Ongoing screening; and (7) Truncating screening. During Step 6 and/or 7, you may also choose to optimize your team, by shifting some members to other review stages (e.g., full-text screening, data extraction).

**Conclusion:**

Artificial intelligence and, more specifically, AML are well-developed tools for title and abstract screening and can be integrated into the screening process in several ways. Regardless of the method chosen, transparent reporting of these methods is critical for future studies evaluating artificial intelligence and AML.

**Supplementary Information:**

The online version contains supplementary material available at 10.1186/s12874-021-01451-2.

## Glossary of terms in the context of systematic reviews

**Table:** 

**Active machine-learning**: An iterative process whereby the accuracy of the predictions made by the algorithm is improved through interaction with reviewers as they screen additional records [1].	
**Artificial intelligence**: Simulation of human intelligence in machines that are programmed to think like humans and mimic their actions [2].	
**Level 2 automation**: Tools enable workflow prioritization, e.g., prioritization of relevant abstracts; however, this does not reduce the work time for reviewers on the task but does allow for compression of the calendar time of the entire process [3].	
**Level 4 automation**: Tools perform tasks to eliminate the need for human participation in the task altogether, e.g., fully automated article screening decision about relevance made by the automated system [3].	
**Reviewer compatibility**: A setting in systematic review software that allows you to restrict certain users from screening each other’s records. For example, if Reviewer A and Reviewer B are restricted, if Reviewer A screens a record, it will be removed from the list of records for Reviewer B. You may also assign a certain range of reference identification numbers to reviewers. These settings will ensure that two junior reviewers will not screen the same records.	
**Stakeholders**: A person or group with a vested interest in a particular clinical decision and the evidence that supports that decision. For example, local government, or health insurance groups [4].	
**Training set**: A set of records which contribute to the active machine-learning algorithm.	

## Introduction

 Systematic reviews (SRs) are one type of review in the spectrum of knowledge synthesis products. Other examples include overview of reviews, rapid reviews, and scoping reviews [5]. SRs are the cornerstone of evidence-based medicine [6], supporting clinical decision-making such as through use in guidelines, and informing policy decisions [7]. However, SRs are time-consuming and there is growing demand by stakeholders to produce evidence more quickly, while maintaining robust methods.

In performing a SR, several methods may be employed to screen records at the title and abstract level. In alignment with current recommendations for SR conduct, screening is typically performed by two reviewers working independently, with conflicts resolved through discussion, or alternatively by consultation of a third person when consensus cannot be achieved [8]. As this approach can be especially time-consuming in the presence of large citation yields, other methods are used, for example the liberal accelerated screening strategy, in which a second reviewer screens those excluded by the first reviewer [9]. Single-reviewer screening can also be used, although when empirically evaluated, this approach may miss many relevant studies [10–13]. In one study, single reviewer screening missed an average of 13% of relevant studies among 24,942 screening decisions [11]. Other alternatives include first performing title-only screening [14, 15], and using more experienced (or expert) reviewers [16].

Artificial intelligence (AI) and more specifically, active machine-learning (AML) have emerged during the past decade as an area of focus to expedite the performance of knowledge syntheses, and may offer potential value both in terms of time saved and costs averted [17]. Teams producing knowledge syntheses products (e.g., SRs) may use this feature to gain efficiencies in their work to meet the needs for rapid evidence generation. AI has recently been introduced in several SR software applications, such as Abstrackr [18], DistillerSR® [19], EPPI-Reviewer [20], Pico Portal [21], Rayyan [22], RobotAnalyst [23], and SWIFTActive-Screener [24], with a comprehensive list available at SR Toolbox (http://systematicreviewtools.com/index.php). These tools use active machine-learning (AML) to re-order (or prioritize) citations to be displayed in order from most likely to be relevant to least likely, a level 2 automation for human-computer interactions [3]. The interest in using AI to support the conduct of SRs and other types of knowledge syntheses (e.g., rapid reviews, scoping reviews) is gaining momentum. Several studies have been published since 2015 using and evaluating the use of AI and prioritized screening, many with encouraging results [10, 25–37]. For example, to identify 95% of the studies included at the title and abstract level, studies have reported a reduction in the number of records that need to be screened of 40% [32] and 47.1% [34].

The development and interest in the use of AI and AML in the context of knowledge syntheses may be due to: (1) the rapid increase in research publications that has caused SR teams to experience large screening burden while conducting reviews; (2) general demand by knowledge users for shorter timelines and lower cost reviews; (3) increased demand for updating reviews and producing living reviews, which require efficiencies in the review process [38]; and (4) the push for evidence-informed decision-making, especially during emergencies (e.g., COVID-19). The use of AI may offer a multitude of potential gains relevant to stakeholders and research teams that include more timely production/delivery of preliminary findings, more efficient use of team member skills, and reduction of screening burden. To facilitate the achievement of such gains, user friendly automation technologies must be seamlessly set up with minimal disruption to processes and resources [3]. Our experiences and interactions with other research teams in the field have suggested there remains interest in the sharing of perspectives with regard to the implementation of such tools into workflow planning of knowledge synthesis.

A review by O’Mara-Eves in 2015 reported that several studies evaluated machine-learning for reducing the in workload for screening records, but noted that there is little overlap between the outcomes (e.g., recall of 95% vs retrieving all relevant studies), making it difficult to conclude which approach is best [1]. More recent studies have generally concluded that full automation (level 4 automation; see Glossary of Terms) performs poorly, while semi-automation (level 2 automation) may be more reliable [10, 30, 33, 34]. Although AI is not currently suitable to fully replace humans in title and abstract screening, there is value to be gained from AI use and some basic principles for teams who produce knowledge synthesis products to adopt are needed.

### Objective

With no current consensus on how to best use AI for study selection, and several studies published in the area performance of AI and AML [26, 27, 30–32, 34], many researchers are may be interested by the premise, but are uncertain as to its validity and means for operationalization. As some of the barriers to adoption of new technologies are the challenges in set-up [3], we have provided different situations and considerations for knowledge synthesis teams to consider when using AI for title and abstract screening while conducting reviews.

## Methods

### Research informing this guidance

We present suggestions for implementation of AML during citation screening for knowledge syntheses based upon recent retrospective and prospective assessments we have conducted in our program of research in knowledge synthesis.

#### Retrospective evaluation of AML

In 2020 we presented findings from a retrospective evaluation of the AML tool for citation screening available in DistillerSR® (Evidence Partners Incorporated, Ottawa, Canada), a software tool for systematic review management, to measure its performance in terms of accuracy (to identify potentially relevant citations) and potential for time savings, and also to develop empirical experience in its use to further guide our work flow for future systematic reviews [34]. In this work, we sought to assess the impact of AML when targeting a 95% true recall rate in terms of identification of studies that progressed to Level 2 full text screening during the initial systematic review. This work measured a variety of parameters of relevance to systematic reviewers including screening hours saved (compared to a traditional screening approach) and ‘missed’ citations included in the final review. Detailed findings are described elsewhere [34], and we have also provided a tabular summary of key review characteristics and AML-related outcomes in Additional file 1. Briefly, in inspecting findings across the 10 systematic reviews that were evaluated, data were consistently supportive of strong accuracy in terms of highlighting relevant citations, as well as achieving researcher-relevant reduction in screening burden. Across the 10 reviews, in no case was a citation selected for final inclusion in any of the reviews missed for progression to full text screening in the current exercise. We point readers to the related manuscript of findings for additional detail.

#### Prospective implementation of ALM

In addition to the above retrospective investigation, since adoption of DistillerSR®‘s AML features as part of the workflow within the research unit of team members CH and BH, the strategies described in this guidance were assessed in terms of their benefits and challenges in the context of recent knowledge syntheses related to the benefits of different primary care models for long-term care homes [39, 40], interventions to manage chronic pain in those with comorbid mental health conditions [41], interventions to reduce the risk of acute pain transitioning to chronic pain [41], the health effects of cannabis consumed by older adults [42], and interventions for management of methamphetamine disorder [43]; we provide additional information regarding these reviews in Additional file 1 to provide context for readers. Authors CH and BH have overseen the implementation of AML in these reviews, monitored their benefits and challenges, and continually refined their approach. The guidance presented has been discussed collectively amongst our co-authorship team in our efforts to enhance our use of AML in our work. Our intent in sharing these steps is to inform others seeking to implement these methods in their workflow, and we hope to pursue future discussions to continually develop this process.

## Guidance

**Fig. 1:**
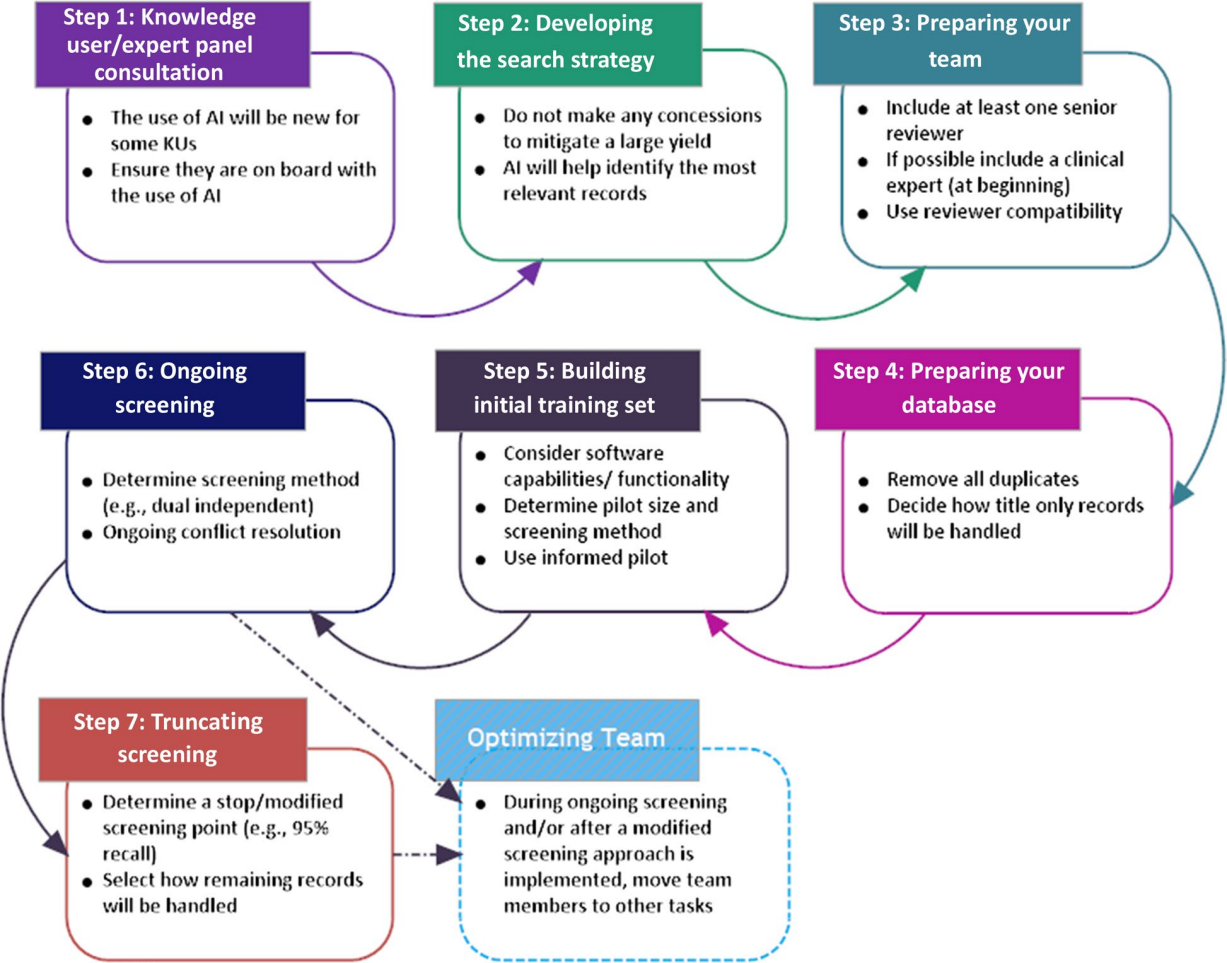
Seven-step approach to integrating active machine-learning into title/abstract screening

Similar to the stages of conducting a SR, we developed a seven-step framework, (Fig. 1) which provides an overview of the steps for the use of prioritized screening. This framework was based on the logical steps from communicating with stakeholders (Step 1), developing a search strategy (Step 2), and the steps leading up to making a decision on when to stop screening and what modified screening approach may be used (Step 7). As previously stated, there are several software packages which now incorporate AI tools to help with title/abstract screening. We have tried to consider the array of available tools in this document to the extent possible, as some required a paid subscription. As many members of the authorship team primarily use DistillerSR, many examples or features described may be specific to DistillerSR and may or may not be available in these other applications.

### Step 1: Knowledge user/expert panel consultation

The use of AI is relatively new when conducting knowledge syntheses, and knowledge users and stakeholders of reviews may not be familiar with how AI can be integrated into the review process. Even if a review team is confident in the application of AI-informed screening while conducting a review, it is important to discuss this with stakeholders before AI is used, especially if a stop/ modified screening approach will be implemented as there is a small chance that relevant records may be missed (this is further discussed below). If the team’s intention is that all records will be screened, but AI will be used to identify the most relevant records first, it is less important to discuss this with stakeholders, as it will not impact which references will be screened. This may, however, be of interest to knowledge users, as they may have access to preliminary information and findings sooner. Any known limitations of the proposed AI approaches should also be clearly identified here to stakeholders, if known.

### Step 2: Developing the search strategy

When developing search strategies, review teams often make concessions to the search during its development to balance screening volume with the risk of missing relevant studies. However, these concessions in the search strategy often removes records from the results (i.e., search yield) solely based on MeSH headings and keywords associated with the record. These omitted records are never accounted for in preparing the final report of a review. With the availability of AML, it is recommended to perform the highest quality search strategy, regardless of yield. During screening, the AML will prioritize records based on relative probability of inclusion, and any records not screened (if a stop-screening approach is used) are on file and could be accessed at any time.

### Step 3: Preparing the research team

It is common to construct a review team composed of junior and senior reviewers, which can be based on prior experience with SRs, performance during pilot screening, and/or content expertise. Depending on the complexity of the review question and/or the makeup of the review team members, it is recommended that reviewer compatibility (setting a restriction in the software to ensure that certain reviewers will not be able to screen each other’s records; see Glossary of Terms) be implemented, if supported by the software package being used to manage the review. This may decrease the number of conflicts, or the number of studies that are incorrectly included/excluded by junior reviewers because of inexperience, rather than an unclear abstract. This is important as records that are incorrectly included or excluded will reduce the accuracy of the AML. If a review team is large and there are fewer senior reviewers to junior reviewers, this might create additional work for the senior reviewers, so team planning and workload of reviewers should be appropriately considered. It is also important to have a person administering the review (e.g., senior reviewer, software specialist) who understands the implications of using the AI features, how to use them appropriately, and how to determine if the AI or AML are not optimal for a particular review (e.g., a review that is answering several questions may not be optimal for AML).

### Step 4: Preparing your database of retrieved records

Duplicate records and title-only records (i.e., records without an abstract) retrieved may contribute to suboptimal AML, as their presence in a database of citations opens to possibility for human reviewers to make conflicting judgements; however, we have provided suggestions for approaches that can be used to mitigate this issue.

#### Duplicate records

Best practice for SRs involves searching a minimum of two bibliographic databases (e.g., Medline, Embase) [8]. As a record may be indexed in multiple databases, typically a number of duplicate records are identified and need to be removed prior to screening. Deduplication will reduce the screening burden and will also lessen the chance that the same title/abstract is screened more than once, which may increase the chance for conflicting decisions. Conflicting decisions on a duplicate record will impact the accuracy of the AML.

#### Title-only records

Records that have titles and no abstracts are often returned in the search results for any knowledge synthesis. Such records tend to be more difficult to screen, as there is often limited information in the title to determine relevance and inclusion status can only be determined with the full-text article. The best strategy for handling these records is not currently known, but several options can be implemented:

1. As title-only screening has been shown to have high recall [12, 13], screen these records in the order they appear (based on the re-ranking algorithm), with the knowledge that they may be incorrectly informing the machine-learning algorithm;
2. Have a response option such as ‘unclear – title-only record’ which stores these records in a neutral response category (if software supports this option). These neutral responses should not inform the machine-learning algorithm and can be re-screened later when the impact of the decisions will be less influential on the machine learning algorithm.
3. Isolate the records temporarily so they do not appear in the list of records for screening. This may be done several ways, depending on the features of the software you are using (e.g. assignment of a neutral tag regarding inclusion status for screening, or by temporarily quarantining the related citations). When the impact of these records will be less influential to the machine learning algorithm, they can be screened.

These options may be helpful for handling of records from clinical trial registries, which are now integrated into online databases. Although some may have an abstract, it is often not structured in the same way as commonly seen for a published study and may be difficult to determine relevance.

### Step 5: Building a high-quality initial training set

Many of the recently introduced AI prioritization tools in SR management software use AML.

There are two ways AML may be used during screening of title/abstract records:

1. To sort records in order of likelihood of inclusion (where likelihood is established based on scores of perceived relevance based on a training set of citations exposed to AML), while still screening all records. While this does not reduce the number of records to be screened, gains can be made as the review team gets access to the most relevant citations faster, and members of the team may be allocated more efficiently to different review stages (e.g., procurement of full-text articles, full text screening, data extraction and risk of bias appraisal), while the records more likely to be excluded can be screened by other members of the review team; and
2. To implement a stop-screening rule or modified screening approach, whereby a decision is made once the AI identifies that a certain threshold has been achieved (e.g., 95% estimated recall). At this threshold, the review team may choose to stop screening the remaining unscreened records or to modify how screening is performed (e.g., changing from dual independent to single screener). These approaches are further discussed below.

Records in a new database will not be shown in a prioritized order, as the AML has not yet ‘learned’ which records should be included or excluded. Reviewer decisions from a small set of records (i.e., the initial training set), whose size will vary depending on which software application is being used, will inform the AML. Once this initial training set is built/screened, the AML is activated, and records are shown to the reviewers in prioritized order. Each additional set of responses (or training sets) contributes to the AML and reshuffles, or prioritizes, the order in which the unscreened records appear to the reviewer. Therefore, the accuracy of each subsequent re-ranking and re-ordering of citations depends on the accuracy of the records already screened and included in the training set. It is particularly important that the initial training set is accurate (i.e., true includes and true excludes are identified), as any errors in screening will ‘teach’ the machine learning algorithm incorrectly.

In the standard approach to SRs, review teams commonly perform a pilot screening exercise on a set number of records to calibrate team member interpretation of the screening question and to expose team members to a sample of the records. This provides the opportunity to not only pilot the screening question(s), but to build a high-quality initial training set prior to application of AI to prioritize. For example, in DistillerSR, the initial training set is built after 2% (minimum of 25 records, maximum of 200 records) of the records are screened (i.e., an include/exclude decision has been made)*.* Therefore, the pilot exercise could be performed on 2% of the records in the database of retrieved records. In Abstrackr, prioritization is run once every 24 h, so this should be considered in project planning. In SWIFT-Active Screener, prioritization first runs after specific conditions are met and then continuously each time 30 references are screened. We suggest the following considerations for implementation of the pilot/initial training set:

- ***Piloting.*** Have two (or more depending on team size) reviewers screen the same set of references independently, with the include- or exclude-decision based on the number of participating reviewers. The amount of references in this pilot set can be either a specific number of records (e.g., 50 records) or a percentage of the total number of citations. For example, in DistillerSR, a database with 7500 records will require 150 records (i.e., 2% of total records) to be screened to create the first training set. Depending on the number of screeners in a review team, they may choose to either all screen the same records (i.e., four reviewers screen the same 150 records) or to split these records between the screeners (e.g., two reviewers each screen 75 records). After these records have been screened by the review team, conflict resolution should be performed. It is possible that after the initial records are piloted that the prioritization tool has not become activated (e.g., you have piloted less than 2% of the total records) or other software requires additional records screened or the timing of prioritization is not immediate (e.g., Abstrackr ranks records once in a 24-h period). Some review teams require a specific agreement level (e.g., kappa of 0.8) to be met before piloting can be considered complete. If this is required, subsequent pilot screening may be required until this level of agreement is achieved.
- ***Reviewer expertise.*** If feasible, it can be beneficial to have an expert reviewer (e.g., clinical or content expert) involved in piloting the initial training set. Experts commonly have a good grasp of the literature and can identify relevant and irrelevant records with high accuracy. This can be an excellent complement to the expertise of other reviewers and help to maximize the training of the AI early on.
- ***Targeted screening to enhance training set.*** It can be highly efficient to conduct a targeted search of the records to build a more *informed* training set. Practically speaking, when developing a grant application or protocol for a SR and gaining expertise in a particular field, some of the relevant studies that will be included in the future review are often identified, whether through identification by participating experts, knowledge brokers, or independent searching by the review lead. Identifying other similar reviews in the area may also offer a list of potentially relevant studies. This may be especially important if the review question is on a condition/disease that is rare, and/or where few included studies may be identified. Identifying these seed articles into the training set early can prove valuable in teaching the AML and should help identify similar citations which may also be relevant.

### Step 6: Ongoing screening

Depending on the software application, the AI prioritization tool will only re-order records that have been fully classified as included or excluded (i.e., no conflict), while others may inform the AML on partially screened records (e.g., SWIFTActive-Screener). The rate at which re-ordering happens varies across currently available SR management software programs. For example, in DistillerSR, after each additional 2% of the records have been fully screened, it creates an *iteration*, which is added to the existing training set, and generates an updated prioritized list based upon all previously screened records; there is also the option to re-rank the records at any time if you do not want to wait for 2% of the records to be screened. In SWIFTActive-Screener, the active learning model is continuously updated during screening, improving its performance with each article reviewed. Currently, Abstrackr has been designed to re-order records once every 24 h. Therefore, if reviewers screen records at a different pace, re-ranking of records will not occur for the faster reviewer until a second reviewer has screened that record, and the prioritization of records may not be optimized for the faster reviewer.

To screen the remaining records, we suggest the following options, in order of methodological robustness. It should be noted that some of the features described below may not be available in all software packages.

- *Dual-independent (best practice).* If the project schedule and timeline allow for it, it is recommended that dual-independent screening be continued, as was used in the pilot training set. However, it is recommended that additional project management be performed. For example, the project lead should implement checks throughout screening at specific intervals (e.g., at the end of each day) to ensure that reviewers are screening records at approximately the same pace to optimize performance and utility of the prioritization tool. Depending on the time availability of the reviewers, establishing daily targets for screening volume may help maintain a common pace across team members. For larger review teams, where time allocation to the project varies for different reviewers due to competing priorities, this may be burdensome and more complex to manage. Additionally, the project lead should suspend screening and have team members resolve their conflicts in cases where conflicts are occurring with some frequency. While this might take some additional time, the time saved by having an accurate training set outweighs the time you will spend screening with an inaccurate model. It might be important to inform the reviewers that records are being displayed in order of likelihood of inclusion, as they may question why they are including so many records, which is not usually the case when screening without prioritization.
- *Liberal accelerated screening*. This requires one reviewer to include a record and two reviewers to exclude a record [9]. As records are being included by one reviewer, the prioritization tool can re-rank the records based on these decisions. The caveat for this option is that over-inclusiveness of records may decrease the accuracy of the machine learning, thereby limiting the gains in efficiency that may be achieved. For records that are in conflict (i.e., the first reviewer excluded the record and the second reviewer included the record), these should be resolved to increase the training set accuracy. This may be done at set intervals (e.g., at the end of each day). If this method is used, it is recommended that the review team is made up of experienced screeners.
- *Single reviewer screening*. You may choose to have one reviewer (e.g., expert, senior reviewer) screen the remaining records. There is a chance for both *false positives* (i.e., inclusion of a record that should have been excluded) and *false negatives* (i.e., exclusion of a record that should have been included) using this option. False positives are less of a concern, as they will be excluded at full-text screening, however, they will impact the accuracy of the training set and prioritization of the remaining records. Although not related to AI and AML, false positive records also contribute to additional procurement costs and full-text screening burden. DistillerSR® includes an AI simulation tool which helps identify potential false positives. False negatives are more concerning, as these would be removed from any further screening. Depending on the software, it is possible to mitigate some of this risk by regularly running an audit of the excluded records. For example, in DistillerSR®, there is an AI audit tool which assigns a prediction score to excluded records with inclusion characteristics and displays this list to the reviewers to double-check exclusion. This may be performed at set intervals (e.g., 5–10% of records, once per day). Resolving incorrect includes and excludes regularly means your reviewers are always screening the most likely includes and will identify these relevant records sooner.

### Step 7: Truncation of screening

Although there is little empirical evidence to support a *modified-screening* or *stop-screening* approach, review teams might choose to stop or modify how they have been screening once a particular threshold has been met. There are several straightforward stopping rules which may be implemented, including stopping once a certain number of irrelevant records are reviewed consecutively (i.e., a heuristic approach) and stopping at a particular point due to time constraints (i.e., pragmatic approach). However, the reliability and accuracy of these methods remains uncertain. There have also been some more complex evaluations to implement a stopping decision [44]. For example, a review team may decide to stop screening once a specific percentage of the predicted relevant references has been identified (e.g., estimated recall of 95%). As not all records would have been screened at this point, the percentage would be based on the *estimated recall*, which may or may not be equal to the *true recall*. Recall is calculated as [True Positives / (True Positives + False Negatives)]. Therefore, if we do not know the value of the True Positives, because we have not screened all records, then we have an estimated recall value. The number of false negatives can be decreased by using the audit tool. There is evidence to support that the estimated recall is in fact a conservative estimate of the true recall [32].

Once the modified/stop-screening criterion has been met, there are several options on what screening method(s) can be used with the remaining records. Table 1 presents the options to screen the remaining records. This list is presented in order from the highest risk of missing a relevant record to least risk. Different approaches may be taken as additional screening has been performed, as the likelihood of inclusion of the remaining references decreases with each iteration.

**Table 1:** Screening options

Approach	Process	Risk	Mitigating risk
1. Stop screening	Change the number of reviews required to 1 and assign the AI tool to exclude the remaining records. If the software does not allow for this, you would leave the remaining records unscreened. There would be no further human screening in this option.	Exclusion of relevant records at title/abstract (i.e., false negatives).	Depending on the threshold that has been used, it may be beneficial to run the AI audit tool^a^ to help identify any false negatives.
2. Single-reviewer screening	Change the include and exclude rules to “1 to include/exclude” and have a single-reviewer screen the remaining records. This may be performed by more than one reviewer, however, only one reviewer will be required to screen any given record.	Over-inclusion of records to be screened at full text (i.e., false positives). Exclusion of relevant records at title/abstract (i.e., false negatives).	Over-inclusion: none Identify false negatives: run AI audit tool^a^
3. Liberal accelerated screening with AI reviewer, with no conflict resolution	Assign the AI reviewer to exclude the remaining records with human reviewers to screen the remaining records using the liberal accelerated approach^b^, with no conflict resolution performed.	Over-inclusion of records to be screened at full text (i.e., false positives). Records in conflict will be ignored by the machine learning algorithm and will not contribute to the prediction scores.	Over-inclusion: none Records in conflict: see approach 4.
4. Liberal accelerated screening with AI reviewer, with conflict resolution	As 3 above, with conflicts resolved. If there is a conflict between the AI reviewer and the human reviewer, a second human reviewer will be required to adjudicate.	Over-inclusion of records to be screened at full text (i.e., false positives). Records in conflict will be ignored by the machine learning algorithm and will not contribute to the prediction scores until conflicts are resolved.	Over-inclusion: none Records in conflict: perform conflict resolution at set intervals (e.g., at the end of each day) so all screened records will contribute to the machine learning.
5. Liberal accelerated screening, no conflict resolution	Change the include rule to “1 to include”, with no conflict resolution performed. Screening will continue with two or more reviewers.	Over-inclusion of records to be screened at full text (i.e., false positives). Records in conflict will be ignored by the machine learning algorithm and will not contribute to the prediction scores.	Over-inclusion: none Records in conflict: see approach 6
6. Liberal accelerated screening, with conflict resolution	As 6 above, with conflicts resolved.	Over-inclusion of records to be screened at full text (i.e., false positives). Records in conflict will be ignored by the machine learning algorithm and will not contribute to the prediction scores until conflicts are resolved.	Over-inclusion: none Records in conflict: perform conflict resolution at set intervals (e.g., at the end of each day) so all screened records will contribute to the machine learning.
7. Dual-independent with AI reviewer	Assign the AI reviewer to exclude the remaining records with human reviewers to screen the remaining records (i.e., dual-independent screening). Another reviewer would be required in cases where the AI reviewer excluded the record and the human reviewer included the record.	Excluding relevant records (i.e., false negatives), as only a single human reviewer is required to exclude (in addition to the AI reviewer). Records in conflict will be ignored by the machine learning algorithm and will not contribute to the prediction scores until conflicts are resolved.	Identify false negatives: run AI audit tool^a^ Records in conflict: perform conflict resolution at set intervals (e.g., at the end of each day) so all screened records will contribute to the machine learning.
8. Dual-independent, assign some reviewers to full-text screening	Not all reviewers may need to continue title and abstract screening. You may choose to move some of the reviewers to perform full-text screening, while keeping a smaller team of reviewers screening the remaining records at title/abstract.	None, although you may need to be strategic on which reviewers are screening title/abstracts.	Keep at least one senior reviewer (based on experience or clinical expertise) to help ensure high-quality include/exclude decisions.

^a^The AI audit tool will identify records that have been given high prediction scores (>0.85) among those that have been excluded

^b^One reviewer required to include and two reviewers required to exclude [9]

## General screening process flow

**Fig. 2:**
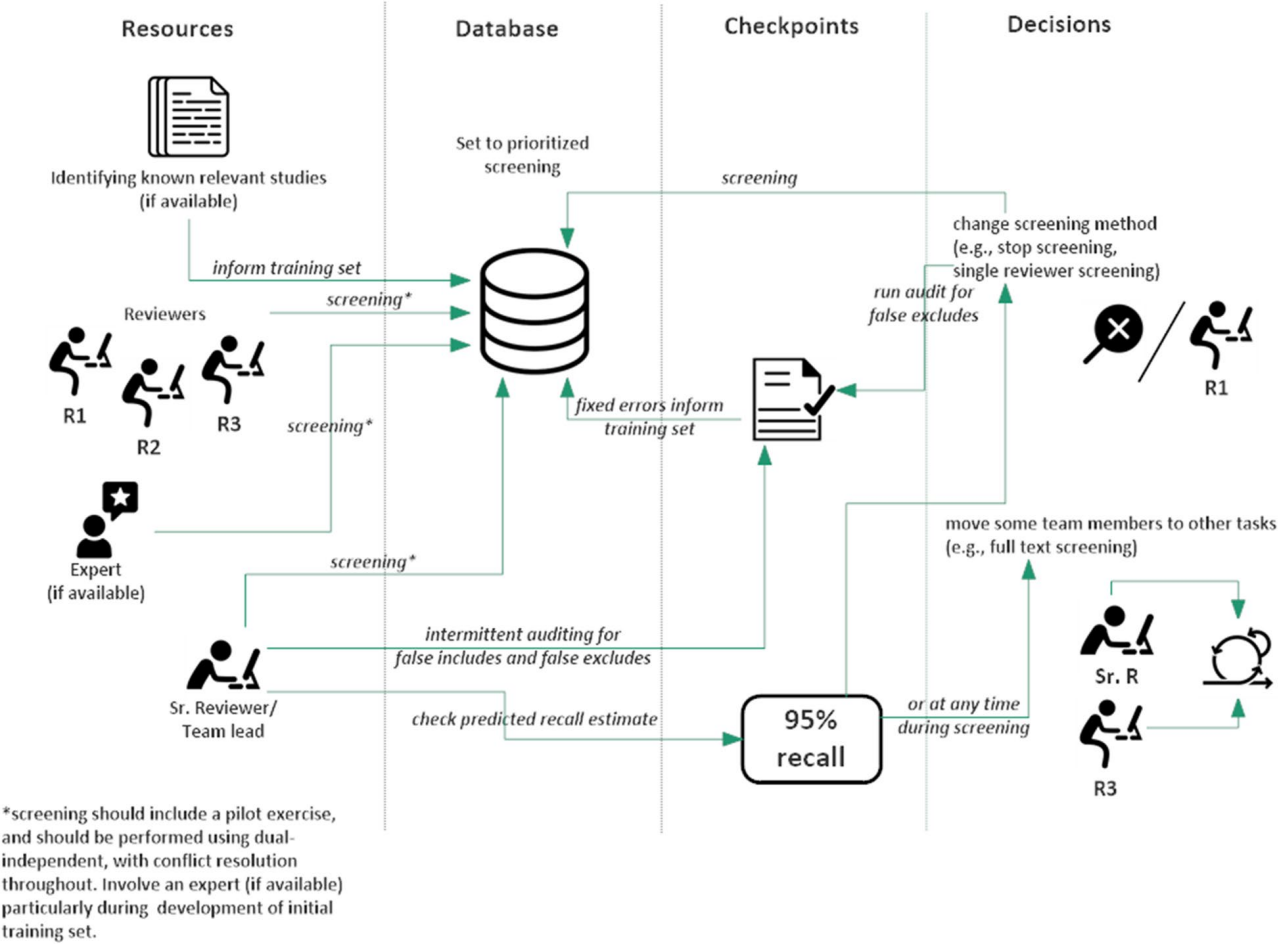
Integration of AI into the overall title and abstract screening process

The previous section described the guidance and considerations from knowledge user consultation through to truncation of screening. We provide here a general screening process flow diagram which provides a pictorial representation of the title and abstract screening process using AML (Fig. 2). As mentioned in the preceding section, some features may not be available in all software applications (e.g., audit), but other processes remain the same.

## Inappropriate use of AI during screening

Review teams might be tempted to develop a training set with a pre-specified set of records (e.g., 200 records), then assign the AI reviewer to have the ability to make include and exclude decisions based on the predicted score (e.g., include those with a score of 0.5 and higher, and exclude the remaining records). Scores assigned by the classifiers may be highly specific to the project and are only useful in relation to the scores of other references. As mentioned earlier, studies that have evaluated this level of automation (level 4) have reported poor performance [10, 30] and this approach should be avoided.

## Discussion

We present guidance for the use of AI and AML during title and abstract screening based on common questions that review teams may encounter while deciding to use (or not use) this approach, and outline effective, lower risk practices for using AI. This work was motivated by our own past hesitation to explore AI methods for work in our field, as well as discussions with our peers who have similarly wondered how to introduce efficiencies from AI into their work while minimizing risk of biases and maximizing an approach that aligns with their current approach. As most research in this area is based on a small number of case studies [30] or small datasets [33], it is important for knowledge synthesis teams to test prioritization tools in their own projects, and we encourage replication to build to the repository of information.

Transparent reporting is critical for any research team conducting primary studies and knowledge syntheses products (e.g., systematic review, scoping review). The Enhancing the QUality and Transparency Of health Research (EQUATOR) network provides researchers with 452 reporting guidelines for all research types, and 43 reporting guidelines specific to SRs/Meta-analyses/Overviews/Health Technology Assessments/Reviews (as of February 2021) [45]. Updates to the Preferred Reporting Items for Systematic Reviews and Meta-Analyses (PRISMA) statement have included requirements for reporting around the use of automation tools with a list of ‘essential elements for systematic reviews using automation tools in the selection process’ [46]; we feel this to be a sign of growing acceptance of the use of AI in the field of knowledge synthesis that should encourage those interested to adopt these tools into their research approach. Transparent and consistent reporting will help determine which title and abstract screening methods were applied when conducting the review, which will allow for replicability, and ultimately allow for conclusions to be made on best approaches and to address concerns of stakeholders [47].

### Implications for future research

Study design of inclusion may impact performance of AML, as RCTs may have better reported abstracts than observational studies, as there is guidance on what should be reported in RCT abstracts [48]. Future research can examine how study design (e.g., RCT vs observation) impact performance, if any. The type of review may also impact performance. For example, an overview of reviews includes SRs as the unit of inclusion, and a scoping review may have a broader scope that a more focused SR. For a review that aims to answer multiple questions, the value of creating separate searches or projects within the software application should be evaluated. In our work, we commonly apply AML separately to citations organized by design, which may be helpful for readers. Our prospective assessments have involved use in large rapid and scoping reviews, and benefits of AML for screening have continued to be considerable.

Stopping or the modifying screening process after the identification of a recall of 95% presented here is the value has that been evaluated in the literature using various software applications. To date, these are based on a small number of reviews. There is an advantage to having a broader number of reviews per primary study and additional evaluative studies to contribute to the overall evidence base. This may contribute to overlapping methodologies and increase the sample size for each methodology evaluated.

### Limitations

One of the difficulties of providing guidance for using AI and AML for title and abstract screening is the rapidly evolving nature of machine learning tools. However, until a time that these tools can fully replace humans, a standard set of methodologies and evaluations will be beneficial to the knowledge synthesis community. Additionally, there are several SR software applications, both freely available and at a cost, which provide different features. Not all guidance and recommendations provided in this manuscript will be applicable to all software applications. It is recommended that users visit the specific websites of these tools to help determine if a particular software contains specific features, as software development may be ongoing and new features made available. We acknowledge that our guidance for use has been based on our own experiences using DistillerSR, a product with which we have considerable expertise through our own use during our past decade of research; we provide the current set of guidance with the objective of helping others based on our own experiences and research.

## Conclusions

AML is a well-developed tool for title and abstract screening and has the potential to reduce the amount of time spent screening titles and abstracts, and may help make optimal use of review team members. There are several ways AI and AML can be integrated into the screening process, and this document has provided a set of recommendations and guidance around its integration. Regardless of the method chosen, transparent reporting of these methods are critical for future studies evaluating AI and AML.

## Supplementary Information

**Additional file 1: Table 1.** Synopsis of Key Findings from Empirical Evaluation of DistillerSR ALM for Level 1 Screening. **Table 2.** Knowledge Syntheses Involving Prospective use of AML in DistillerSR Software.

## Data Availability

Not applicable.
