# Supplementary material for: Guidance for using artificial intelligence for title and abstract screening while conducting knowledge syntheses
Source: BMC Med Res Methodol. 2021 Dec 20;21:285. doi: 10.1186/s12874-021-01451-2 (PMC8686081; doi:10.1186/s12874-021-01451-2)
Supplement: Supplementary file 1 — Additional file 1: Table 1. Synopsis of Key Findings from Empirical Evaluation of DistillerSR ALM for Level 1 Screening. Table 2. Knowledge Syntheses Involving Prospective use of AML in DistillerSR Software. [file 12874_2021_1451_MOESM1_ESM.docx]

**Appendix 1: Additional Information, Retrospective and Prospective Assessments of Active Machine Learning**

**Table 1: Synopsis of Key Findings from Empirical Evaluation of DistillerSR ALM for Level 1 Screening**

***Based upon*:** An evaluation of DistillerSR's machine learning-based prioritization tool for title/abstract screening - impact on reviewer-relevant outcomes. Hamel C, Kelly SE, Thavorn K, Rice DB, Wells GA, Hutton B. BMC Med Res Methodol. 2020 Oct 15;20(1):256. We refer readers to this publication for more detailed description of these data.

| **Systematic review [Protocol reference]** | **Review type**$\boldsymbol{\dagger}$**; eligible designs** | **Intervention type** | **Number of studies at:** | | | **Outcome Measures of**  **AML Benefit** | | |
| --- | --- | --- | --- | --- | --- | --- | --- | --- |
|  |  |  | **Ti/Ab** | **Full-text** | **Final⁑** | **Level 1 hours saved (95% recall)** | **% of relevant L1 citations not identified at 95% recall** | **Final included studies missed** |
| Interventions for hot flashes [1] | Effectiveness review (NMA);  RCTs only | Pharmacological and behavioural therapies | 2569 | 451  (17.6%) | 38  (1.48%) | 27.9 | 6.2% | 0 |
| Interventions for opioid use disorder [2] | Effectiveness review (NMA);  RCTs only | Psychosocial therapies | 16282 | 984  (6.0%) | 71  (0.44%) | 196.7 | 5.4% | 0 |
| Interventions for Meniere’s disease [3] | Effectiveness (NMA);  RCTs only | Pharmacological therapies, surgical interventions | 2889 | 332  (11.5%) | 23  (0.80%) | 28.7 | 5.7% | 0 |
| ALK inhibitors for non-small cell lung cancer [CRD42017077046] [4] | Effectiveness review (NMA);  RCTs & observational | Pharmacological therapies | 3145 | 795  (25.3%) | 13  (0.40%) | 21.9 | 5.9% | 0 |
| Influenza vaccination during pregnancy [<https://osf.io/xey2k/>] | Effectiveness review;  RCTs & observational | Pharmacological intervention | 8278 | 395  (4.8%) | 104 (1.26%) | 87.6 | 4.8% | 0 |
| E-cigarettes for smoking cessation [5] | Effectiveness review;  RCTs & observational | E-cigarette | 2250 | 881  (39.2%) | 14  (0.62%) | 11.3 | 6.0% | 0 |
| Omalizumab for atopic asthma and chronic idiopathic urticaria  [CRD42018082211] [6] | Effectiveness and safety review;  RCTs & observational | Pharmacological intervention | 3265 | 482  (14.8%) | 12  (0.36%) | 29.6 | 5.4% | 0 |
| Adult depression screening [7] | Effectiveness review;  RCTs only | Screening intervention | 4174 | 126  (3.0%) | 1  (0.02%) | 35.8 | 4.8% | 0 |
| Antiretroviral drugs and implementation strategies for HIV pre-exposure prophylaxis [CRD42017073014] | Effectiveness & Etiology review;  RCTs & observational | Pharmacological and behavioural therapies | 4502 | 1184  (26.3%) | 46  (1.02%) | 30.0 | 5.4% | 0 |
| Sugar sweetened beverages and adverse health outcomes [8,9] ‡ | Effectiveness & Etiology review;  RCTs & observational | Interventional/ behavioural and exposure to SSBs | 22309 | 4993  (22.4%) | 127 (0.57%) | 158.6 | 5.2% | 0 |

NMA: Network Meta-analysis; RCT: Randomized Controlled Trial; SR: Systematic Review

† Based on typology classifications from Munn 2018 [10]

‡ Protocol published as two separate reviews, with references screened in one project silo.

⁑ The final included studies does not always reflect the study designs that were included in the protocol, as decisions may have been made during the process of the review. However, the study designs reflect those that were included in the search strategy and screened at title/abstract.

**Table 2: Knowledge Syntheses Involving Prospective use of AML in DistillerSR Software**

| **First Author; Review Type; Funder; status** | **Review Topic** | **Eligibility Criteria Synopsis** | **Size of Review (initial search and # of included articles)** |
| --- | --- | --- | --- |
| Hamel C et al [11,12];  Scoping Review; SPOR Evidence Alliance;  published | Models of provider care in long-term care | - Population: Residents of long-term care homes with any condition. Palliative care was limited to within long-term care homes; hospice settings, residential homes, and skilled nursing facilities were excluded. - Intervention/Comparators: Models of provider care, or interventions delivered to facility, staff, and residents in long-term care homes. Resident exposure to staffing levels/mix of staffing was of most interest. This included studies evaluating different approaches/ arrangements of staffing. Studies evaluating access to or direct services provided by relevant care providers to residents were also included. Provision of care could be delivered by any of the following: medical care; direct patient care; allied health care. Studies involving comparisons between different models of provider care and studies wherein different models were compared over time (e.g. interrupted time series) were included. - Outcomes: quality of life (QoL); Quality of care (QoC); health outcomes (e.g., mortality; chronic disease management indicators; etc); healthcare worker (HCW) stress; burnout or quality of work-life if reported along with a relevant primary outcome. - Study design: Randomized controlled trials, non-randomized controlled trials, and quasi-experimental study designs (e.g., controlled before/after studies [CBA]; interrupted time series [ITS]) were of primary interest. Cross-sectional studies, case-control studies, case reports and qualitative literature were excluded. - Time frame: 2010 - 2020 | 7,574 citations screened;  366 included after full text screening |
| Rice D et al [13]; Rapid Review; Canadian Institutes of Health Research;  technical report available (manuscript in preparation) | Interventions to manage chronic pain in individuals with concurrent mental health or substance use disorders | - Population: Individuals diagnosed with chronic pain (pain lasting at least three months) who have a concurrent mental health or substance use diagnosis - Intervention/Comparator: Pharmacological, psychological, physical (e.g., physiotherapy), and self-management interventions, including biopsychosocial / multidisciplinary / interprofessional approaches (e.g., stepped care, multidisciplinary team pain clinics). - Outcomes: no restrictions. - Study design: Clinical practice guidelines (CPGs), best practice recommendations, overviews of guidelines, overviews of reviews/umbrella reviews, meta-analyses (MAs), network meta-analyses (NMAs) - Language: English and French language documents; CPGs from Canada, USA, UK, or Australia, or from a European or international body - Time frame: published between 2015-2020 | 8,676 citations screened;  7 included after full text screening |
| Wolfe D et al https [13]; Rapid Review; Canadian Institutes of Health Research; Technical report available (manuscript in preparation) | Interventions to reduce the risk of transitioning from acute pain to chronic pain | - Population: Individuals with acute pain (less than three-month duration) due to surgery or trauma/injury as well as patients anticipated to have post-surgical pain at a future date - Intervention/Comparator: Interventions to reduce the risk of transition from acute to chronic pain, including assessment of risk factors, timely access to a multidisciplinary team post-onset of pain, pharmacologic interventions (e.g., selective serotonin reuptake inhibitors), psychological(e.g., cognitive behavioural therapy), physical (e.g., pre-hab), and self-management/ behavioural interventions - Outcomes: transition to chronic pain, which may have been measured as the presence or intensity of pain at three months or beyond the pain-inciting event; composite outcomes with a pain component; continuous/ persistent opioid use beyond three months of initial prescription. - Study design: Clinical practice guidelines (CPGs), best practice recommendations, overviews of guidelines, overviews of reviews/umbrella reviews, meta-analyses (MAs), network meta-analyses (NMAs) - Language: English and French language documents; CPGs from Canada, USA, UK, or Australia, or from a European or international body - Time frame: published between 2015-2020 | 10,188 citations screened;  44 included after full text screening |
| Wolfe D et al [14]; Scoping Review; Canadian Institutes of Health Research; manuscript in preparation | Health effects of cannabis consumption in older adults | - Population: Older adults (e.g., at least 80% of participants must have been > 50 years of age, or if age data were not reported, >80% of participants must have had end-stage cancer, Alzheimer’s disease, dementia, or Parkinson’s disease, or an age- or condition-stratified analysis must have been reported for an age group over 50 years or one of the identified conditions). Current cannabis use was of interest, defined as use within the past year. - Interventions/exposures: medical or non-medical cannabis of any type, with any mode of consumption, or dosage. - Outcomes: both beneficial and harmful cannabis-related effects on physical and mental health and behaviour were of interest. - Study design: Study design: overviews of reviews, systematic reviews, RCTs, non-randomized studies (NRSs), and observational studies. - Time frame: from the year 2000 | 31,393 citations screened;  133 included after full text screening |
| Hamel C et al [15]; Scoping Review; Canadian Institutes of Health Research; manuscript in preparation | Psychosocial and pharmacologic interventions for methamphetamine disorder | - Population: Individuals with methamphetamine use disorder or problematic methamphetamine use (per DSM, ICD-10, or earlier ICD versions) - Interventions / Comparators: both psychosocial and pharmacologic interventions were of interest. ***Psychosocial interventions:*** cognitive behavioral therapy, motivational enhancement therapy, contingency management, motivational interviewing, community reinforcement approach, support programs, acceptance and commitment therapy, dialectical behavioral therapy, mindfulness therapy, other psychosocial treatments, and combinations of these strategies. ***Pharmacologic interventions:*** dopamine agonists (e.g., amantadine), psychostimulants (e.g., dextroamphetamine, methylphenidate), GABA (e.g., baclofen, acamprosate, gabapentin), antipsychotics (e.g. aripiprazole), opioid antagonists (e.g., naltrexone), antidepressants (e.g. sertraline, bupropion, mirtazapine, imipramine), cognitive enhancers (e.g., rivastigmine, galantamine, varenicline, modafinil), topiramate, oxytocin, vaccines, other pharmacologics, and combinations of pharmacologics. - Outcomes: Changes in methamphetamine (and other substance) use, study retention, treatment retention, acceptability of intervention, mental and physical health, self-efficacy, withdrawal symptoms, legal/employment outcomes, harms - Study design: systematic reviews, guidelines, randomized controlled trials | 14,129 citations screened;  61 included after full text screening |

# References for Table 1 and 2

[1] Hutton B, Yazdi F, Bordeleau L, Morgan S, Cameron C, Kanji S, et al. Comparison of physical interventions, behavioral interventions, natural health products, and pharmacologics to manage hot flashes in patients with breast or prostate cancer: protocol for a systematic review incorporating network meta-analyses. Syst Rev 2015;4:114. https://doi.org/10.1186/s13643-015-0099-y.

[2] Rice DB, Hutton B, Poulin P, Sproule BA, Wolfe D, Moher D, et al. Evaluating comparative effectiveness of psychosocial interventions for persons receiving opioid agonist therapy for opioid use disorder: protocol for a systematic review. BMJ Open 2018;8:e023902. https://doi.org/10.1136/bmjopen-2018-023902.

[3] Ahmadzai N, Cheng W, Wolfe D, Bonaparte J, Schramm D, Fitzpatrick E, et al. Pharmacologic and surgical therapies for patients with Meniere’s disease: a protocol for a systematic review and meta-analysis. Syst Rev 2019;8:341. https://doi.org/10.1186/s13643-019-1195-1.

[4] Elliott J, Bai Z, Hsieh S-C, Kelly SE, Chen L, Skidmore B, et al. ALK inhibitors for non-small cell lung cancer: A systematic review and network meta-analysis. PLOS ONE 2020;15:e0229179. https://doi.org/10.1371/journal.pone.0229179.

[5] Hersi M, Traversy G, Thombs BD, Beck A, Skidmore B, Groulx S, et al. Effectiveness of stop smoking interventions among adults: protocol for an overview of systematic reviews and an updated systematic review. Syst Rev 2019;8:28. https://doi.org/10.1186/s13643-018-0928-x.

[6] Johnston A, Smith C, Zheng C, Aaron SD, Kelly SE, Skidmore B, et al. Influence of prolonged treatment with omalizumab on the development of solid epithelial cancer in patients with atopic asthma and chronic idiopathic urticaria: A systematic review and meta-analysis. Clin Exp Allergy 2019;49:1291–305. https://doi.org/10.1111/cea.13457.

[7] Hamel C, Lang E, Morissette K, Beck A, Stevens A, Skidmore B, et al. Screening for depression in women during pregnancy or the first year postpartum and in the general adult population: a protocol for two systematic reviews to update a guideline of the Canadian Task Force on Preventive Health Care. Syst Rev 2019;8:27. https://doi.org/10.1186/s13643-018-0930-3.

[8] Hamel C, Stevens A, Singh K, Ansari MT, Myers E, Ziegler P, et al. Do sugar-sweetened beverages cause adverse health outcomes in adults? A systematic review protocol. Syst Rev 2014;3:108. https://doi.org/10.1186/2046-4053-3-108.

[9] Stevens A, Hamel C, Singh K, Ansari MT, Myers E, Ziegler P, et al. Do sugar-sweetened beverages cause adverse health outcomes in children? A systematic review protocol. Syst Rev 2014;3:96. https://doi.org/10.1186/2046-4053-3-96.

[10] Munn Z, Stern C, Aromataris E, Lockwood C, Jordan Z. What kind of systematic review should I conduct? A proposed typology and guidance for systematic reviewers in the medical and health sciences. BMC Med Res Methodol 2018;18:5. https://doi.org/10.1186/s12874-017-0468-4.

[11] Hamel C, Garritty C, Hersi M, Butler C, Esmaeilisaraji L, Rice D, et al. Effective models of provider care in long-term care homes: a rapid scoping review. Ottawa: Ottawa Hospital Research Insitute; 2020.

[12] Hamel C, Garritty C, Hersi M, Butler C, Esmaeilisaraji L, Rice D, et al. Models of provider care in long-term care: A rapid scoping review. PLoS One 2021;16:e0254527. https://doi.org/10.1371/journal.pone.0254527.

[13] Rice D, Wolfe D, Garritty C, Hersi M, Esmaeilisaraji L, Butler C, et al. Best Practice in Pain Management: Rapid Reviews of Guidelines and Knowledge Syntheses. Ottawa: Ottawa Hospital Research Insitute; 2020.

[14] Wolfe D, Corace K, Rice D, Smith A, Kanji S, Conn D, et al. Effects of medical and non-medical cannabis use in older adults: protocol for a scoping review. BMJ Open 2020;10:e034301. https://doi.org/10.1136/bmjopen-2019-034301.

[15] Hamel C, Corace K, Hersi M, Rice D, Willows M, Macpherson P, et al. Psychosocial and pharmacologic interventions for methamphetamine addiction: protocol for a scoping review of the literature. Syst Rev 2020;9:245. https://doi.org/10.1186/s13643-020-01499-z.
